# Development and validation of four one-step real-time RT-LAMP assays for specific detection of each dengue virus serotype

**DOI:** 10.1371/journal.pntd.0006381

**Published:** 2018-05-29

**Authors:** Benjamin Lopez-Jimena, Michaël Bekaert, Mohammed Bakheit, Sieghard Frischmann, Pranav Patel, Etienne Simon-Loriere, Louis Lambrechts, Veasna Duong, Philippe Dussart, Graham Harold, Cheikh Fall, Oumar Faye, Amadou Alpha Sall, Manfred Weidmann

**Affiliations:** 1 Institute of Aquaculture, University of Stirling, Stirling, Scotland, United Kingdom; 2 MAST Diagnostica GmbH, Reinfeld, Germany; 3 Robert Koch Institute, Centre for biological security 1 (ZBS1), Berlin, Germany; 4 Functional Genetics of Infectious Diseases Unit, Department of Genomes and Genetics, Institut Pasteur, Paris, France; 5 Centre National de la Recherche Scientifique, Unité de Recherche Associée, Paris, France; 6 Insect-Virus Interactions Group, Department of Genomes and Genetics, Institut Pasteur, Paris, France; 7 Virology Unit, Institut Pasteur du Cambodge, Institut Pasteur International Network, Phnom Penh, Cambodia; 8 Arbovirus and viral haemorrhagic fever unit, Institut Pasteur de Dakar, Institut Pasteur International Network, Dakar, Senegal; Centers for Disease Control and Prevention, UNITED STATES

## Abstract

**Background:**

4 one-step, real-time, reverse transcription loop-mediated isothermal amplification (RT-LAMP) assays were developed for the detection of dengue virus (DENV) serotypes by considering 2,056 full genome DENV sequences. DENV1 and DENV2 RT-LAMP assays were validated with 31 blood and 11 serum samples from Tanzania, Senegal, Sudan and Mauritania. DENV3 and DENV4 RT-LAMP assays were validated with 25 serum samples from Cambodia

**Methodology/Principal findings:**

4 final reaction primer mixes were obtained by using a combination of Principal Component Analysis of the full DENV genome sequences, and LAMP primer design based on sequence alignments using the LAVA software. These mixes contained 14 (DENV1), 12 (DENV2), 8 (DENV3) and 3 (DENV4) LAMP primer sets. The assays were evaluated with an External Quality Assessment panel from Quality Control for Molecular Diagnostics. The assays were serotype-specific and did not cross-detect with other flaviviruses. The limits of detection, with 95% probability, were 22 (DENV1), 542 (DENV2), 197 (DENV3) and 641 (DENV4) RNA molecules, and 100% reproducibility in the assays was obtained with up to 10^2^ (DENV1) and 10^3^ RNA molecules (DENV2, DENV3 and DENV4). Validation of the DENV2 assay with blood samples from Tanzania resulted in 23 samples detected by RT-LAMP, demonstrating that the assay is 100% specific and 95.8% sensitive (positive predictive value of 100% and a negative predictive value of 85.7%). All serum samples from Senegal, Sudan and Mauritania were detected and 3 untyped as DENV1. The sensitivity of RT-LAMP for DENV4 samples from Cambodia did not quite match qRT-PCR.

**Conclusions/Significance:**

We have shown a novel approach to design LAMP primers that makes use of fast growing sequence databases. The DENV1 and DENV2 assays were validated with viral RNA extracted clinical samples, showing very good performance parameters.

## Author summary

The co-existence of several dengue virus (DENV) serotypes within the same location and/or individuals as well as a single mosquito being able to carry multiple DENV serotypes highlight the necessity of specific diagnostic tools capable of detect and serotype DENV strains circulating worldwide. In addition, these methodologies must be highly sensitive in order to detect the genome at low levels (i.e., before the onset of clinical symptoms) and not cross-detect other flaviviruses. Isothermal amplification methods (such as reverse transcription loop-mediated isothermal amplification, RT-LAMP) are affordable for laboratories with limited resources and do not need expensive equipment. Because of the increasing number of publicly available full DENV genome sequences, traditional primer design tools are not able to handle such huge amount of information. Therefore, to be able to cover all the diversity documented, we developed 4 complicated oligonucleotide mixes for the individual detection of DENV1-4 serotypes by RT-LAMP. This approach combined Principal Component Analysis, phylogenetic analysis and LAVA algorithm. Our assays are specific and do not cross-react with other arboviruses and DNA pathogens included in this study, they are sensitive and have been validated with samples from Tanzania, Senegal, Sudan, Mauritania and Cambodia, showing very good performance parameters.

## Introduction

Dengue is a worldwide public health concern annually affecting more than 100 million people in tropical and subtropical areas [1, 2]. It is caused by dengue virus (DENV), the most common vector-borne viral pathogen of humans, transmitted by mosquitoes of the *Aedes* genus (primarily *A*. *aegypti* and to a lesser extent *A*. *albopictus*), as previously reviewed [3]. DENV infection in humans results in a broad spectrum of disease manifestations, ranging from self-limiting, acute febrile illness (dengue fever) to more severe forms of the disease (dengue haemorrhagic fever and dengue shock syndrome), which may lead to death [4]. In 2013, the annual global incidence was estimated close to 390 million DENV infections, which was more than three times the dengue burden estimate of the World Health Organization [2].

DENV is an enveloped virus (genus *Flavivirus*, family *Flaviviridae*) with a genome that consists of a single-stranded, positive-sense RNA molecule of about 11 kb in length. The DENV genome encodes three structural proteins (C, capsid; prM, pre-membrane, and E, envelope) at the N terminus and seven non-structural (NS) proteins (NS1, NS2a, NS2b, NS3, NS4a, NS4b and NS5) [5, 6]. This virus is classified into four phylogenetically related and loosely antigenically distinct serotypes (DENV1, DENV2, DENV3 and DENV4), each of which contains phylogenetically different genotypes [7–9].

DENV outbreaks between 2006 and 2013, in India, Vietnam, Solomon Islands, Myanmar, China, Singapore, Malaysia and Portugal [10–14], highlight the necessity of rapid virus detection to identify DENV as the cause of an outbreak, in order to manage and control virus spread in infrastructure poor urban, peri-urban and rural settings. Notably, routine detection of DENV in children who are often asymptomatic carriers could improve outbreak control [15]. A first vaccine has recently been licensed for the prevention of dengue, which aims to reduce the number of hospitalizations per year, being approved for people aged between 9 to 45 years [16].

Traditional virus isolation is time-consuming, requires experienced staff, costly facilities and equipment and needs more than seven days to complete the assay [17, 18]. IgM- and IgG-capture enzyme-linked immunosorbent assay (ELISA) are most widely used but some degree of cross-reactivity against other flaviviruses is usually observed and this method is not useful when antibody titers are not sufficiently high (febrile viremic phase) [19]. Molecular amplification techniques to detect DENV RNA (RT-PCR, quantitative RT-PCR—qRT-PCR), which have emerged as a new standard, have a quick turnaround time and can distinguish DENV serotypes [20–26]. However, these techniques require sophisticated equipment and experienced staff, making them unpractical for laboratories with limited resources.

Loop-mediated isothermal amplification (LAMP) has the potential to substitute PCR-based methods because of its simplicity, rapidity, specificity, sensitivity and cost-effectiveness, as no special equipment is needed (just a heating block or water bath capable to maintain a constant temperature between 60°C to 65°C) [27–29]. Reactions can be visualised by monitoring either the turbidity in a photometer or the fluorescence in a fluorimeter, by visual inspection under UV lamp when using an intercalating dye or by colour change [8, 28–36].

Previously reported reverse transcription LAMP (RT-LAMP) assays for DENV target the 3’ untranslated region (UTR) [8, 30, 32, 34, 37], whilst other detect a fragment of the C-prM region [33], a conserved region of the NS1 [36], or regions of NS2A (DENV1), NS4A (DENV3), NS4A (DENV2) and the 3’ UTR (DENV4) [38]. In all cases information about the primer design is limited as only one sequence per serotype or reference sequences were considered or it is not clearly detailed how the sequence alignment was carried out or how many sequences were included in the design. An initial screen of all published DENV RT-LAMP detection amplicons quickly revealed that all of them fail to cover the documented sequence variation. To improve DENV RT-LAMP design we used the LAMP Assay Versatile Analysis (LAVA) algorithm [39] which solves the limitations of existing LAMP primer-designing programs by allowing designs based on large multiple sequence alignments. Our LAMP design is based on 2,056 whole-genome DENV sequences covering DENV strains from 2004 to 2014 and yielded 4 one-step, real-time RT-LAMP assays to specifically detect each DENV serotype.

## Materials and methods

### Ethics statement

Ethical approval for retrospective use of the anonymized samples in diagnostic development research was available: Tanzania samples (Ethikkommission Basel in Switzerland, Institutional Review Board of the Ifakara Health Institute and National Institute for Medical Research Review Board in Tanzania), IPD and IPC samples (Ministry of Health of Senegal and National Ethics Committee for Health Research of Cambodia, respectively).

### Viral RNA, patient samples and RNA extraction

Virus material: DENV isolates were provided and tested at the Institut Pasteur in Paris (Table 1). TriReagent extracts from flavivirus culture supernatants were provided by M. Weidmann. Inactivated strains ATCC VR-344 (DENV1), ATCC VR-345 (DENV2), ATCC VR-1256 (DENV3) and ATCC-1257 (DENV4) were provided by ENIVD / Robert Koch Institute. An inactivated Zika virus strain (ZIKV, H/PF/2013) was provided by Prof. Xavier de Lamballerie (Unité des Virus Emergents, Marseille, France).

**Table 1:** RNA samples used in this study. Cross-specificity and cross-detection results.

Provided by	Pathogen	Strains (Serotype)	RT-LAMP protocols			
			DENV1	DENV2	DENV3	DENV4
Robert Koch Institute^a^	DENV	ATCC VR-344 (D1)	**+**	-	-	-
		ATCC VR-345 (D2)	-	+	-	-
		ATCC VR-1256 (D3)	-	-	+	-
		ATCC VR-1257 (D4)	-	-	-	+
Institut Pasteur Paris^b^	DENV	KDH0026A (D1)	+	-	-	-
		KDH0002A (D1)	+	-	-	-
		KDH0030A (D1)	+	-	-	-
		KDH0032A (D1)	+	-	-	-
		30173/10 (D1)	+	-	-	-
		30520/09 (D1)	+	-	-	-
		DJOM2.9.12 (D1)	+	-	-	-
		R0712259 (D2)	-	+	-	-
		DJ.OS.1.7.12 (D2)	-	+	-	-
		DJ.MO.1.7.12 (D2)	-	+	-	-
		DJWA1.7.12 (D3)	-	-	+	-
		KDH0012A (D3)	-	-	+	-
		KDH0014A (D3)	-	-	+	-
		KDH0010A (D3)	+	-	+	-
		VIMFH4 (D4)	+	-	-	+
University of Stirling^c^	DENV	DEN1/T081117 (D1)	+	-	-	-
	YFV	YFV/T090109	-	-	-	-
	WNV	WNV P2 24.07.08	-	-	-	-
	NTAV	Ntaya P3 DPP 8.8.13	-	-	-	-
Unité des Virus Emergents^d^	ZIKV	H/PF/2013	-	-	-	-
MAST Diagnostica GmbH^e^	*S*. Typhi	ST	-	-	-	-
	*S*. Paratyphi	SP	-	-	-	-
	*S*. *pneumoniae*	Spn5	-	-	-	-
	*P*. *falciparum*	3D7	-	-	-	-

^a^ Dr Pranav Patel, Robert Koch Institute, Centre for biological security 1 (ZBS1), Berlin, Germany

^b^ Dr Anavaj Sakuntabhai (Functional Genetics of Infectious Diseases Unit) and Dr Louis Lambrechts (Department of Genomes and Genetics). Isolates from clinical samples in Myanmar, Cambodia, Thailand and Gabon between 2007 and 2010. VIMFH4 was isolated in 1976.

^c^ Prof. Manfred Weidmann, Institute of Aquaculture, University of Stirling, United Kingdom.

^d^ Prof. Xavier de Lamballerie, Unité des Virus Emergents, Marseille, France.

^e^ Dr Mohammed Bakheit, MAST Diagnostica GmbH, Reinfeld, Germany.

An External Quality Assessment (EQA) 2015 panel was provided by QCMD (Quality Control for Molecular Diagnostics, Glasgow, UK) including ten unknown samples (15–01 to 15–10).

Patient samples: We used RNA extracts of 31 blood samples collected during a fever study in Tanzania, 2013 (Table 2) provided by the Swiss Tropical and Public Health Institute in Basel, Switzerland. These samples included 24 DENV qRT-PCR positive, 2 DENV positive (not characterized by qRT-PCR) and 5 negative samples. In addition, a negative sample from MAST Diagnostica GmbH (Reinfeld, Germany) was included. RNA extracts of 11 DENV qRT-PCR serum samples from Senegal, Sudan and Mauritania collected in November-December 2014 by the Institut Pasteur in Dakar (IPD), Senegal (Table 3) were tested by qRT-PCR and LAMP in Dakar. Additionally serum samples from Cambodia collected through the National Dengue Surveillance System [40] were tested. RNA was extracted and air-dried using pre-dried RNAstable 1.5 mL microfuge tubes (Biomatrica, USA) from 13 DENV3 and 12 DENV4 samples, collected by the Institut Pasteur du Cambodge (IPC) in 2004–2006 and between 2008 and 2014, respectively. Samples were shipped at ambient temperature. Moreover, samples were tested by qRT-PCR before shipment and after receipt and reconstitution in molecular grade water. Overall the qRT-PCR C_T_ deviation was in a range of 0.8 C_T_. Five μL RNA of each sample were used per reaction.

**Table 2:** Blood samples used in this study, analysed by real-time RT-PCR and RT-LAMP.

Pathogen	Patient ID	C_T_ values	RNA from 50 μL blood	RNA from 100 μL blood		
			Initial T_T_ values (min)	Current T_T_ values (min)		
				Mean	SD	Positives/total replicates
**DENV2**	**1341**	26.11	37			
	**1371**	25.89	38			
	**1226**	24.38	40			
	**1284**	27.36	43			
	**1329**	27.51	44			
	**1343**	27.93	49			
	**1430**	27.63	50			
	**1478**	27.52	50			
	**1217**	25.53	50			
	**1207**	27.24	52			
	**1472**	26.57	53			
	**1337**	28.13	56			
	**1473**	29.13	81	73.9	0.3	2/3
	**1342**	28.41	81	62.4	2.1	3/3
	**1365**	21.57	84	55.0	0.0	3/3
	**1352**	26.27	87	77.0	10.4	3/3
	**1321**	23.81	89	58.5	2.4	3/3
	**3053**	NT^a^	-^b^	-		0/3
	**3062**	NT	-	-		0/3
	**1232**	28.78	-	-		0/3
	**1363**	28.16	-	61.7	3.4	3/3
	**1270**	26.79	-	67.8	3.3	3/3
	**1273**	26.71	-	68.4		1/3
	**1488**	26.45	-	72.2	12.2	3/3
	**1257**	26.15	-	64.1	1.7	3/3
	**1241**	24.27	-	70.0		1/3
**Non-DENV2 (negative samples)**	**1479**	-	NT	-		0/3
	**1090**	-	NT	-		0/3
	**1025**	-	NT	-		0/3
	**1126**	-	NT	-		0/3
	**1158**	-	NT	-		0/3
**S**	**S**	NT	NT	-		0/3

^a^ NT: non-tested.

^b^ negative result

**Table 3:** RNAs tested from samples collected by the Institut Pasteur in Dakar (DENV 1, 2) in 2014, and Institut Pasteur du Cambodge (DENV3, 4).

IPD/IPC number	C_T_ values*	T_T_ values (min)#	Origin	Serotype
**267197**	25.89	20	Senegal	1§
**267196**	26.17	20–21	Senegal	1
**267174**	27.22	20	Mauritania	1§
**267175**	29.79	21–22	Mauritania	1§
**267150**	26.15	28–29	Senegal	2
**267267**	27.82	30–31	Senegal	2
**267234**	33.22	38–45	Senegal	2
**267219**	36.52	36–43	Senegal	2
**267186**	37.62	40–45	Senegal	2
**267213**	38.09	32–45	Sudan	2
**267207**	38.48	39–45	Senegal	2
**P1212131**	24.78	-	Cambodia	3
**Q0427132**	25.66	59.36	Cambodia	3
**R0104070**	27.73	15.75	Cambodia	3
**R0104072**	28.87	-	Cambodia	3
**P0921232**	32.01	-	Cambodia	3
**R0104074**	32.25	-	Cambodia	3
**P0913209**	32.55	-	Cambodia	3
**Q0427138**	34.21	57.03	Cambodia	3
**P1111026**	34.8	-	Cambodia	3
**Q0531203**	36.05	-	Cambodia	3
**Q0427140**	36.06	-	Cambodia	3
**Q0529123**	37.24	-	Cambodia	3
**R0302118**	39.33	-	Cambodia	3
**T0423100**	28.17	41–48	Cambodia	4
**W1019304**	28.52	40	Cambodia	4
**Z0603308**	29.7	-	Cambodia	4
**Z0722323**	30.45	36–37	Cambodia	4
**Y0807311**	30.66	40–43	Cambodia	4
**Z0603310**	31.51	36	Cambodia	4
**Z0617306**	31.62	-	Cambodia	4
**T0408073**	31.71	46	Cambodia	4
**Y0521311**	31.73	33	Cambodia	4
**Y0731302**	32.73	33	Cambodia	4
**Z0713303**	-	-	Cambodia	4
**U0927345**	-	41	Cambodia	4

* C_T_ (qRT-PCR) as tested at IPD immediately before testing RT-LAMP (DENV1, DENV2); C_T_ as tested immediately before shipment by IPC (DENV3), C_T_ as tested on arrival of shipment (DENV4). C_T_ values are listed incremental per DENV type.

# T_T_ ranges of LAMP results: triplicates for DENV1 and DENV2, duplicates for DENV3 and DENV4. A single T_T_ result represents one positive out of 2 (DENV3, DENV4).

§ Serotype determined by RT-LAMP.

### RNA extraction

RNA extractions were carried out using the RNeasy mini (DENV strains from Robert Koch Institute, QCMD samples) (QIAGEN, Crawley, West Sussex, UK) and the QIAamp Viral RNA mini (DENV samples from IPD and IPC and ZIKV strain from Unité des Virus Emergents) (QIAGEN, Courtaboeuf, France) kits. TriReagent extracts were processed according to the manufacturer’s extraction protocol (Sigma-Aldrich, Dorset, UK).

RNA extraction of the clinical samples from Tanzania was initially performed from 50 μL whole blood using a trial version of a nucleic acid isolation system equivalent to the protocol established for the MagSi-gDNA blood kit (MagnaMedics, Geleen, The Netherlands). RNA was eluted in 190 μL elution buffer, and 5 μL per sample were used for each RT-LAMP reaction. Additionally, an improved trial version of the MagnaMedics system for nucleic acid isolation, starting from 100 μL whole blood and eluting the RNA in 100 μL elution buffer, using 5 μL per sample for each RT-LAMP reaction, was used. RNA was extracted from the clinical samples from Senegal using the QIAamp Viral RNA mini kit.

### DENV qRT-PCR and nested PCR

A DENV RNA standard was transcribed from the DENV 3’ UTR region, ligated into pCRII and evaluated as previously described [41]. DEN FP and DEN P were as described with the probe now tagged 5’-FAM / BBQ-3’ but an adapted reverse primer DEN RP2 (5’-CTGHRGAGACAGCAGGATCTCTG-3’) as described [42]. DENV qRT-PCR was performed using the Light Cycler 480 Master Hydrolysis Probes (Roche, Mannheim, Germany) in a 20-μL reaction volume containing 1x LightCycler 480 RNA Master Hydrolysis Probes, 3.25 mM activator Mn(OAc)_2_, 500 nM primers DEN FP and DEN RP2, 200 nM probe DEN P, and 1 μL RNA template on the LightCycler 2.0 (Roche), as follows: reverse transcription for 3 min at 63°C, activation for 30 s at 95°C, followed by 45 cycles consisting of amplification for 5 s at 95°C and 15 s at 60°C and a final cooling step of 40 s at 40°C. Analysis of the reactions was conducted using LightCycler software version 4.1.1.21 (Roche).

The Institut Pasteur in Dakar performed a qRT-PCR [43], using the ABI7500 Fast Real-time PCR System (Applied Biosystems, Foster City, CA). An RT-PCR assay, which simultaneously detects the 4 DENV serotypes, followed by a nested PCR, that specifically detects each DENV serotype, were used [20].

### LAMP primer design

A two-step approach was used. First, all available sequences of DENV1 to 4 were downloaded from the NCBI database. Searches were limited to the samples collected between 2004 and 2014. All sequences were then aligned (for each serotype) using GramAlign v3.0 [44], and diversity was assessed using the *glPCA* module of R/adegenet v1.4.1 [45]. Finally, based on the Principal Component Analysis (PCA) and phylogenetic tree (Neighbor-Joining tree using the R/ape 3.2 package), the sequences were manually split into different clusters in order to maximise the region of sequence identity. LAMP DNA signatures for each cluster (and all combinations to minimise the number of primer sets) were designed using a modified version [https://github.com/pseudogene/lava-dna] of LAVA [39] applying the *loose* parameters set for DENV1-3 and the *standard* parameter set for DENV4. Full scripts and methods are available on GitHub at https://github.com/pseudogene/lamp-denv.

All the designed sets of primers were first checked for primer dimerisation with the VisualOMP version 7.8.42.0 (DNA Software, Ann Arbor, MI). In addition, primer combinations for each of the DENV assays were tested for primer dimerisation by comparing amplification signals in positive and negative controls.

### One-step real-time RT-LAMP

RT-LAMP reactions were run at 64°C using either an ESE-Quant TubeScanner (QIAGEN Lake Constance GmbH, Stockach, Germany) or Genie II (Optigene, Horsham, UK), in a final reaction volume of 25 μL. The Genie II device displays the annealing curve for specificity after the reaction has finished, by melting curve analysis from 98°C to 80°C (0.05°C/s).

Four RT-LAMP assays were developed, one for each DENV serotype (S1 File). Each reaction consisted of 1x RM Trehalose, 6 mM MgSO_4_, 5% polyethylene glycol (PEG), 1 μL fluorochrome dye (FD), 8 U *Bst* 2.0 DNA Polymerase (New England BioLabs, Hitchin, Herts, UK), 10 U Transcriptor Reverse Transcriptase (Roche) and 1 μL template (DENV RNA or H_2_O as negative control). For each primer set per RT-LAMP assay, the final concentrations was as follows: 50 nM F3, 50 nM B3, 400 nM FIP, 400 nM BIP, 200 nM FLOOP, 200 nM BLOOP. Before adding the *Bst* 2.0 DNA Polymerase, Transcriptor Reverse Transcriptase and template, mixes were incubated at 95°C for 5 min to melt any primer multi-mers and cooled immediately on ice for 5 min. Reaction times vary for each RT-LAMP protocol, running for 45 min (DENV1), 90 min (DENV2), 75 min (DENV3) and 50 min (DENV4).

RM Trehalose, MgSO_4_, PEG and FD were supplied by MAST Diagnostica GmbH.

### Sensitivity of the RT-LAMP protocols

Sensitivity analysis was performed in the ESE-Quant TubeScanner (QIAGEN). Ten-fold dilutions of viral DENV RNA samples (ATCC VR-344 (DENV1), ATCC VR-345 (DENV2), ATCC VR-1256 (DENV3) and ATCC VR-1257 (DENV4)), quantified by qRT-PCR, were used to analyse the sensitivity of the developed RT-LAMP assays (range from 10^4^−10^5^ to 10 molecules/μL) and 1 μL per dilution was added to the RT-LAMP reaction. The complete RNA standard was tested in eight separate runs. The values obtained were subjected to probit analysis (Statgraphics plus v5.1, Statistical Graphics Corp., Princeton, NJ) and the limit of detection at 95% probability for each RT-LAMP assay was obtained.

### Cross-specificity and cross-detection tests

Cross-specificity tests for the four RT-LAMP assays were carried out at the Institut Pasteur (Paris) using the QuantStudio 12K Flex Real-Time PCR System, and results were analysed with the software QuantStudio 12K Flex v1.2.2. (Applied Biosystems, Carlsbad, CA). Each of the RT-LAMP assays was tested using 1 μL RNA extracted from the DENV strains described in Table 1. Cross detection of other flaviviruses, ZIKV, Yellow fever virus (YFV), West Nile virus (WNV) and Ntaya virus (NTAV), was analysed using the Genie II (Optigene) at the University of Stirling.

The RT-LAMP assays were also tested against several DNA pathogens (*Salmonella* Typhi, *S*. Paratyphi, *Streptococcus pneumoniae* and *Plasmodium falciparum*). DNA samples were provided by MAST Diagnostica GmbH.

The performance of the RT-LAMP assays (sensitivity and specificity) was additionally evaluated using the 2015 DENV EQA panel provided by QCMD. Results obtained from QCMD refer to 8 core and 2 educational samples. Core samples are those needed to assess the performance from the regulatory point of view and educational samples are additional samples related to questions such as limit of detection or specificity.

### Evaluation of the RT-LAMP assays with clinical samples

We used 31 blood samples from a fever study in Tanzania, 2013 (Table 2). Twenty-six samples had been confirmed as DENV2 positive by the Swiss Tropical and Public Health Institute (Basel, Switzerland) (2 of them were not tested by qRT-PCR). Aliquots of these blood samples were sent to MAST Diagnostica GmbH and stored at -20°C until RNA extraction was performed using the Magnamedics kit trial version. RNA samples were stored at -80°C. RT-LAMP reactions were run in the TubeScanner TS2 (QIAGEN), using 5 μL RNA of each sample per reaction.

The samples at IPD were analysed by both qRT-PCR [43], and the DENV1 and DENV2 RT-LAMP assays (in triplicates) in an ABI7500 Fast Real-time PCR system (Applied Biosystems), using 5 μL RNA of each sample per reaction.

Sensitivity, specificity, positive predictive value (PPV) and negative predictive value (NPV) were obtained for the DENV2 RT-LAMP developed when compared against the results obtained by qRT-PCR.

## Results

### Quantification of DENV RNA by absolute one-step qRT-PCR

The RNA standard was tested 3 times and similar crossing point (CP) values were obtained for the different dilutions from 10^7^ to 10^3^ RNA molecules detected (S1 Fig), showing an efficiency (E = 10^−1/slope^—1) of 0.99 ± 0.04 (mean ± standard deviation, SD). Quantification of DENV1-4 RNA extracted from inactivated isolates ATCC VR-344 (DENV1), ATCC VR-345 (DENV2), ATCC VR-1256 (DENV3) and ATCC VR-1257 (DENV4) (Table 1) ranged from 6.9x10^4^–9.4x10^4^ (DENV1), 4x10^5^–5.3x10^5^ (DENV2), 1.5x10^5^ - 3x10^5^ (DENV3), and 1.8x10^5^–2.7x10^5^ (DENV4) RNA molecules/μL.

### LAMP primer design and evaluation

**Fig 1:**
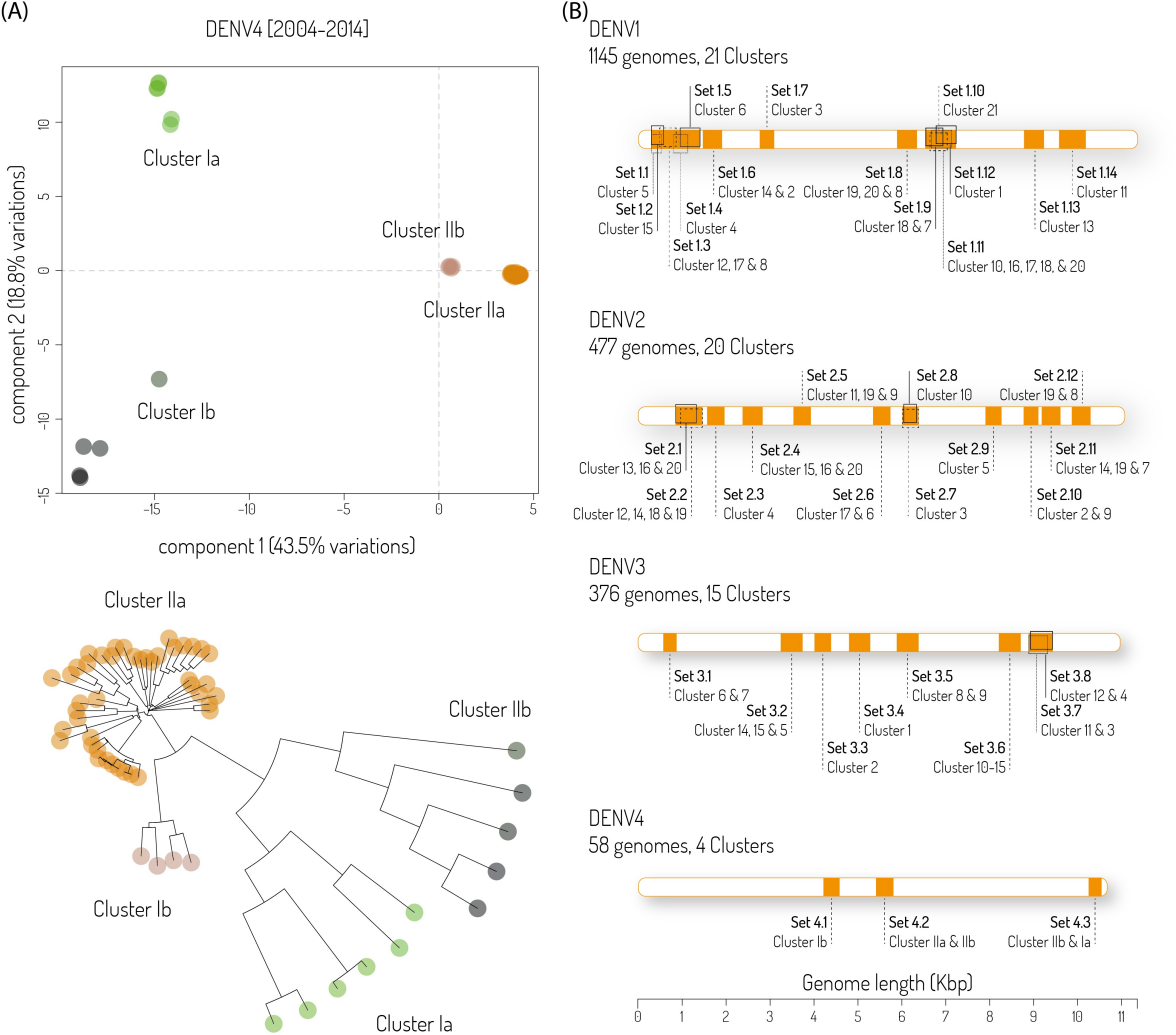
LAMP primer design. (A) PCA and phylogenetic clustering of 58 DENV4 genomes. Four subgroups were necessary to describe all genotypes found (variation explained by first, second and third principal component, 43.5%, 18.8% and 5.9% respectively). (B) Location of all primer sets used for each DENV serotype. Genomes/clusters concerned are also indicated.

In total 1,145, 477, 376 and 58 genomic sequences were retrieved from the NCBI database for DENV1, DENV2, DENV3 and DENV4, respectively. Each serotype dataset was split into 4 to 21 clusters (Fig 1A and S2–S4 Figs), allowing for the LAVA algorithm to design LAMP primer sets, and was executed for each group separately as well as for all possible combinations of the groups.

**Fig 2:**
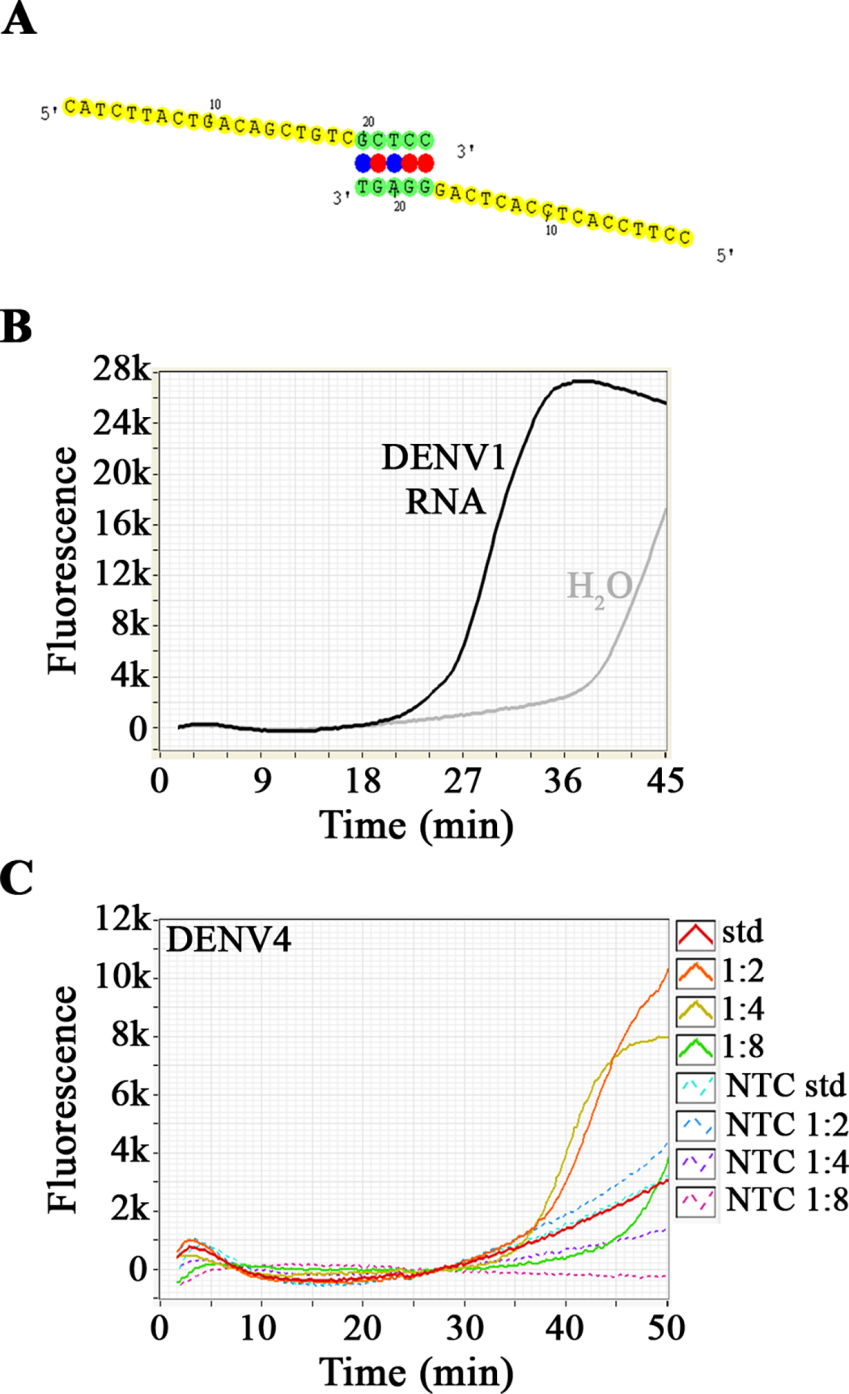
Dimerisation and primer concentration. (A) Example of dimerisation detected by Visual OMP software. (B) Dimerisation detected in no template control during an RT-LAMP reaction. Black line reaction with RNA, grey line: NTC. (C) 2-fold dilution of the primer sets used in the DENV4 RT-LAMP. Continuous lines represent the reactions with RNA, discontinuous lines refer to NTC.

Sets of primers that showed dimerisation when analysed with VisualOMP (DNA Software, Ann Arbor, MI) were discarded (Fig 2A). Remaining sets where sequentially combined and tested by RT-LAMP to discard cases of primer dimerisation, visualised by the non-specific amplification signal (intercalating dye) in the no template control (NTC) (Fig 2B). The final primer sets are described in Fig 1B and S1–S4 Tables and consist of 84 (14 amplicons, DENV1), 72 (12 amplicons, DENV2), 48 (8 amplicons, DENV3) and 18 (3 amplicons, DENV4) primers.

When combining the amplicon primer sets for each RT-LAMP assay, amplification was not observed when using published standard LAMP primer concentrations for each primer set: 0.2 μM F3, 0.2 μM B3, 1.6 μM FIP, 1.6 μM BIP, 0.8 μM FLOOP and 0.8 μM BLOOP. To determine the concentration window of the complicated primer mix, a 2-fold dilution series of the above primer mix was used. Amplification yielding the best possible detection without amplification in the NTC was achieved at a dilution of 1:4 (50 nM F3, 50 nM B3, 400 nM FIP, 400 nM BIP, 200 nM FLOOP and 200 nM BLOOP, Fig 2C).

### Cross-specificity and cross-detection tests

**Fig 3:**
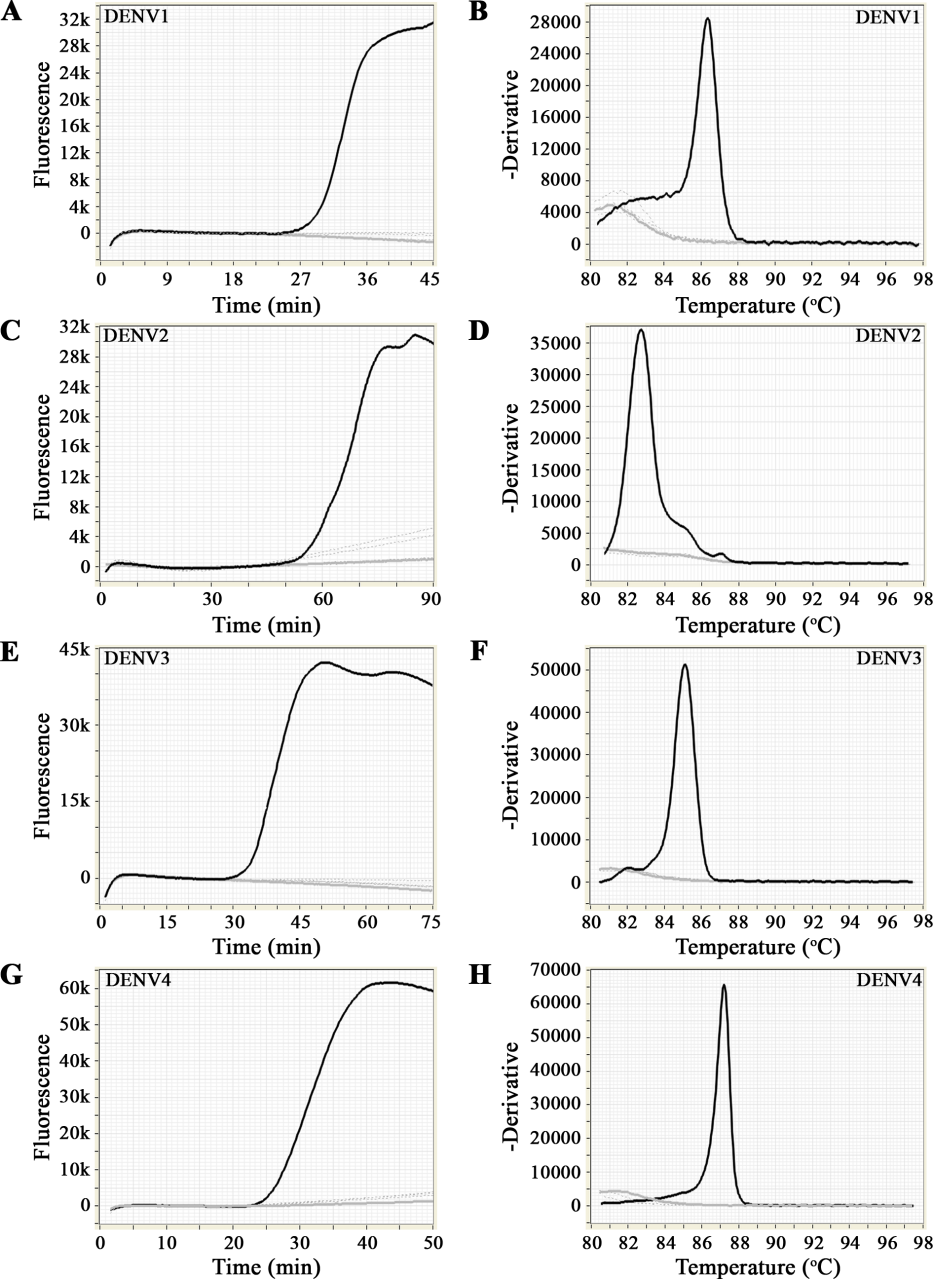
Cross-detection assays to confirm the specificity of the RT-LAMP protocols to detect DENV RNA (black line). There was no amplification of other flaviviruses RNA (discontinuous grey lines) or in the NTC (continuous grey line). (A), (C), (E) and (G) show the amplification profiles for the RT-LAMP reaction. (B), (D), (F) and (H) show the annealing curve for specificity.

**Fig 4:**
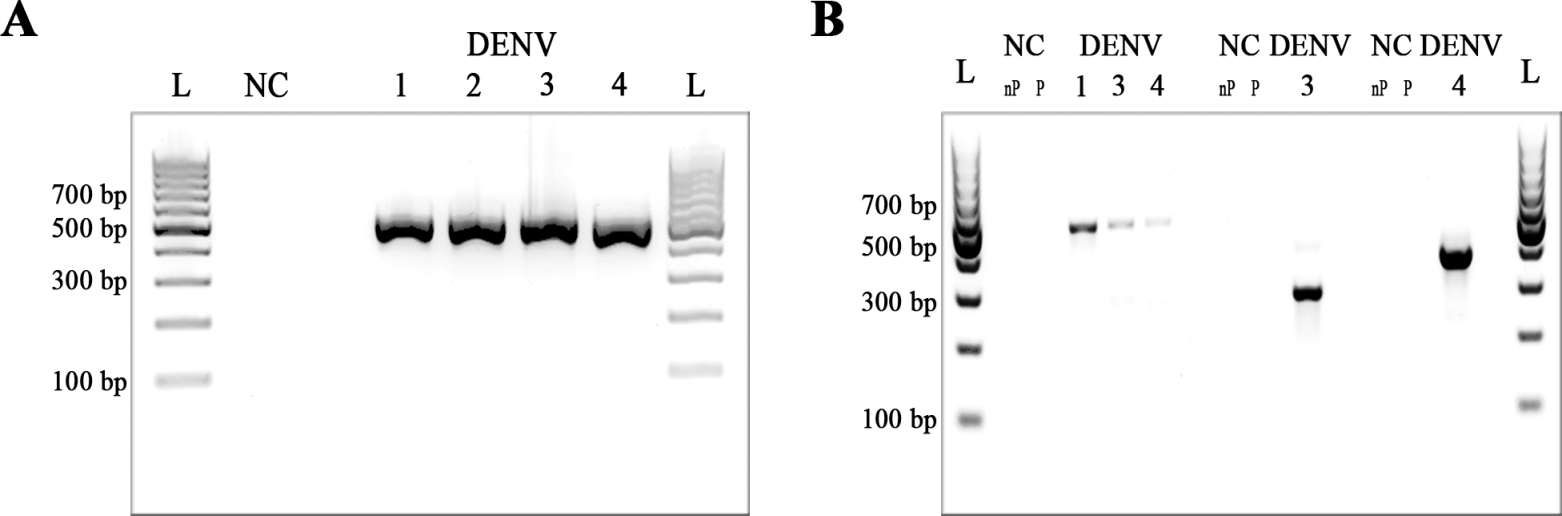
Detection of DENV strains by RT-PCR and nested PCR. (A) RT-PCR using D1 and D2 primers. (B) Serotype-specific nested PCR using D1/TS1, D1/TS3 and D1/TS4 primers to detect DENV1, DENV3 and DENV4, respectively. L: 100 bp DNA ladder (Thermo Scientific); NC: negative control (H_2_O); 1: KDH0030A (DENV1); 2: DJOS1.7.12 (DENV2); 3: KDH0010A (DENV3); 4: VIMFH4 (DENV4); nP: negative control nested PCR; P: negative control PCR.

Table 1 and Fig 3 show the results of the cross-specificity and cross-detection tests. All DENV cell culture RNA extracts were detected and no amplification was observed in the NTC. The RT-LAMP protocols for DENV2, DENV3 and DENV4 were specific for each respective serotype. The RT-LAMP protocol for DENV1 detected all DENV1 RNA strains, but also scored positive in RNA extracts KDH0010A and VIMFH4 containing RNA extracts from DENV3 and DENV4 isolates, respectively (Table 1). Additional testing of samples KDH0010A and VIMFH4 by nested RT-PCR (Fig 4A and 4B) indicated contamination of the cell cultures samples with DENV1 confirming the RT-LAMP results.

The RNA of other flaviviruses was not cross-detected (Fig 3 and Table 1). Specific amplification was also indicated by a specific single peak temperature in the melting curve analysis (Fig 3B, 3D, 3F and 3H), with mean values ± SD of 85.4 ± 1.1°C (DENV1), 83.1 ± 1.0°C (DENV2), 84.3 ± 0.9°C (DENV3) and 86.4 ± 0.3°C (DENV4). No amplification was observed when DNA from *S*. Typhi, *S*. Paratyphi, *S*. *pneumoniae* and *P*. *falciparum* was used as template in the different RT-LAMP assays (Table 1).

The 2015 DENV EQA panel analysis confirmed that the RT-LAMP assays developed passed 8 core and the 2 educational samples of that panel. Concerning the core samples, 5 positive samples were scored 3/3, and 1 positive sample was detected once (the other 2 samples were negative). Results obtained from the educational samples indicated that 1 sample was detected in the 3 repetitions whilst the other sample was detected in 1/3 repetitions.

### Analytical sensitivity of the RT-LAMP protocols

**Fig 5:**
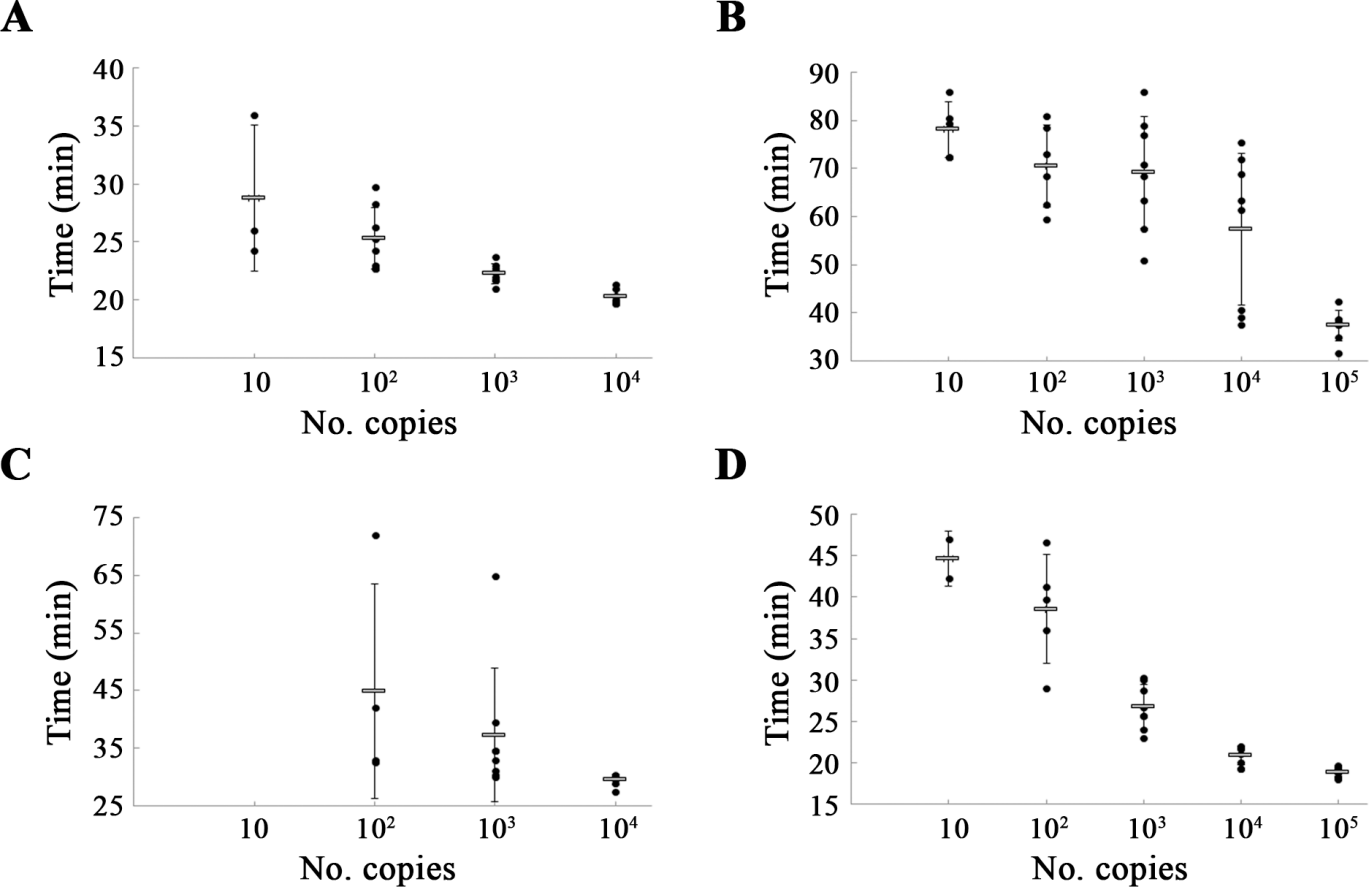
Times (min) of positive detection using serial 10-fold dilutions of DENV RNA. The mean values are represented with a grey bar and error bars indicate the standard deviation. Black dots refer to positive signals of eight independent runs. (A), (B), (C) and (D) represent the plots referring to DENV1, DENV2, DENV3 and DENV4, respectively.

DENV1-4 RNA samples, previously quantified by qRT-PCR, were used to analyse the sensitivity of the developed RT-LAMP assays. RT-LAMP protocols for DENV1, DENV2 and DENV4 detected as few as 10 molecules per reaction, although this amount was only obtained in 3, 5 and 2 of 8 repetitions, respectively, with the following mean times: 28.8 ± 6.3 min (DENV1), 78.2 ± 5.8 min (DENV2) and 44.6 ± 3.3 min (DENV4). RT-LAMP for DENV3 detected as few as 10^2^ molecules, but only in 4 of 8 reactions, at 44.9 ± 18.6 min. The lowest amount of molecules detected in the 8 reactions, showing 100% reproducibility, were 10^2^ (DENV1, mean time of 25.3 ± 2.6 min), and 10^3^ (DENV2, DENV3 and DENV4, mean times of 69.2 ± 11.6 min, 37.2 ± 11.6 min and 26.8 ± 2.7 min, respectively) (Fig 5). Considering 8 independent reactions per protocol developed, the probit analysis revealed that the limit of detection at 95% probability for each RT-LAMP was 22 RNA molecules (DENV1), 542 RNA molecules with a confidence interval from 92 to 3.2x10^13^ RNA molecules (DENV2), 197 RNA molecules (DENV3) and 641 RNA molecules with a confidence interval from 172 to 1.2x10^5^ RNA molecules (DENV4).

### Evaluation of the RT-LAMP with clinical samples

Tables 2 and 3 show the results of the blood and serum samples analyses when using both qRT-PCR and RT-LAMP.

Out of 26 DENV2-infected blood samples 24 scored positive in qRT-PCR with cycle threshold (C_T_) values ranging from 21.57–29.13 (Table 2, column 2). In a first test DENV2 RT-LAMP detected 17/24 (70.8% positive samples) with initial time to positive (T_T_) values between 37 and 89 min (Table 2, column 3).

RNA from 14 samples, including those with initial T_T_ values over 60 min, negative in both RT-LAMP and qRT-PCR, and 6 DENV negative samples (Table 2), were extracted a second time using the optimized MagnaMedics extraction starting from 100 μL sample and yielding enhanced detection.

Five samples with initial T_T_ values from 81–89 min, now tested positive with T_T_ values from 55–77 min. Six samples initially negative by RT-LAMP became positive with T_T_ values of 61.7–72.2 min. Three samples, 1 of which had scored positive in qRT-PCR, remained negative in RT-LAMP. Most RNA samples extracted with the optimized method scored positive in all 3 replicates. One sample was detected 2/3 times, and 2 were detected only once. All negative samples included in these analyses scored negative.

Calculation of the clinical sensitivity and specificity yielded 100% specificity (CI: 0.63–1.00), as no false positives were detected, and a sensitivity of 95.8% (CI: 0.79–1.00) with 23/24 positive samples, a PPV of 1.00 (CI: 0.85–1.00) and NPV of 0.86 (CI: 0.42–1.00).

Table 3 summarises the results obtained with samples collected by the IPD and IPC. All 11 RNA samples from IPD used in this study were analysed in parallel by qRT-PCR and with DENV1 and DENV2 RT-LAMP assays. All scored positive in qRT-PCR (C_T_ 25.89–38.48), 4 samples scored positive in the DENV1 RT-LAMP, and 7 scored positive in the DENV2 RT- LAMP (T_T_ values 20–45 min). Samples 267175, 267197 and 267174 were serotyped as DENV1 with the developed RT-LAMP.

Additionally, of 12 qRT-PCR positive DENV4 samples dried with RNAstable shipped by IPC, 10 tested positive by qRT-PCR after shipment, and 9 were detected by DENV4 LAMP. Of 13 DENV3 samples qRT-PCR positive before shipment, only 1 tested positive by qRT-PCR on arrival and only 3 by RT-LAMP.

## Discussion

Dengue is now prevalent in more than 100 countries of the tropics and subtropics and as DENV continues to spread, all four serotypes co-circulate widely [46–48]. The introduction of new DENV strains continues through travellers moving between dengue-endemic countries [11] and recently the capacity of individual mosquitoes to carry multiple DENV serotypes was described [49], while elsewhere acute simultaneous infection with several DENV serotypes was observed [10].

DENV detection methods include virus culture, which is time consuming [17, 18] as well as ELISA or immunofluorescence methods to detect IgM and IgG which suffer from cross-reactivity to other flaviviruses antibodies and which are only considered valid when antibody titers are sufficiently high [19]. The introduction of NS1 antigen detection has improved the situation and recent studies show a high sensitivity of NS1 detection [50], with some concluding that the combination with IgM detection can outperform PCR [51]. However, its use for routine screening in dengue epidemics is questioned in terms of clinical necessity [52].

For molecular RNA detection, nested PCR [20] and real time PCR-assays [21–26] with high specificity and sensitivity are being used but need expensive and sophisticated thermocyclers and experienced staff. In recent years, isothermal amplification assays have been described, such as RT-LAMP [8, 30, 32–38] and RT-RPA [53, 54]. These assays require less expensive equipment and can be delivered in dried pellet format, making handling easier and amenable to poor infrastructure settings.

Worldwide monitoring and the use of Next Generation Sequencing methods have increased the number of complete DENV genomes sequenced and deposited in GenBank to 2,988 (as of June 2016). It is virtually impossible to use this amount of sequence information to manually align and design amplicons for molecular detection methods. There have been several attempts to create algorithms to derive signature sequences for PCR techniques from sequence datasets or alignments [55, 56]. LAMP amplicons are inherently more challenging to design as they require a minimum of 4 and a maximum of 6 signature sequences. LAVA software was developed to facilitate the determination of signature sequences for LAMP primer design using a set of aligned sequences [39]. The original and modified version of LAVA take into consideration the limitations observed with other primer-design programs (LAMP DESIGNER [http://www.optigene.co.uk/lamp-designer/] and PRIMER EXPLORER [https://primerexplorer.jp/e/], such as preventing the use of extensive alignments or sequences longer than 2,000 nt.

We used this approach to design serotype-specific primers aiming to match all possible DENV strains circulating worldwide, by considering 2,056 available GenBank DENV sequences (2004–2014). This is the greatest difference compared to other previously published RT-LAMP assay designs in which primer design focused on the conserved 3’ UTR, NS1 or C-prM regions but detailed limited information about the DENV sequences used to develop the primers. As the LAMP primers were designed from different clusters of each DENV serotype obtained after PCA and phylogenetic analyses, the individual LAMP amplicons locate to several regions across the DENV genome conserved in these clusters (Fig 1). This allows an overall detection of DENV variability surpassing any other molecular amplification assay. The final amplicons were selected through a combination of *in silico* primer dimer formation assessment (Visual OMP) and *in vitro* assessment by checking amplicons selected in the first step for unspecific amplification in the NTC. A similar methodology has been used to design RT-LAMP primers to detect Chikungunya virus (manuscript submitted to PLoS Neglected Tropical Diseases) and we consider this approach would be suitable for the assay development of other infectious diseases. The final DENV1-4 specific RT-LAMP assays contained 84, 72, 48 and 18 oligonucleotides respectively. The challenge was to find a working concentration of these oligonucleotide mixes, which would allow for sensitive detection. A 2-fold dilution series approach for the individual final primer mix allowed to identify a working concentration window in the dynamic range of these assays. This however came at the cost of run time. In order to increase the reaction speed without losing sensitivity, several combinations of enzymes were tested. We tested the combination of AMV RT (Promega, Southampton, UK) and GspSSD DNA polymerase (Optigene) recommended by others who successfully developed rapid RT-LAMP assays with 10–15 minute run times [57] (Manuguerra personal communication). We also tested *Bst* 3.0 DNA polymerase (New England BioLabs), but found that none offered an advantage over the enzyme combination we used (Transcriptor Reverse Transcriptase and *Bst* 2.0). As a matter of fact, we saw an increased level of unspecific amplification with *Bst* 3.0 DNA polymerase (data non-shown).

Thus currently reaction times range from 45 (DENV1) to 90 minutes (DENV2). This was not correlated with the number of oligonucleotides in the mixture but may reflect the efficiency of the individual primer sets in the mixture detecting the respective standard strains we used for the validation, and the low oligonucleotide concentration. Alternative approaches to evaluate the sensitivity of each RT-LAMP would consist of having either a pool of RNA samples representative for each amplicon included or specific primer sets for each particular DENV strain that would be compared with the primer mixtures included in the developed assays.

We used an RNA standard evaluated by qRT-PCR to quantify viral RNA of DENV1-4. These quantified RNA were then used to test the analytical sensitivity of the 4 individual specific RT-LAMP assays for the detection of each serotype. The analytical sensitivities of the DENV1-4 RT-LAMP assays, as estimated per probit analysis, ranged from 22 to 641 RNA molecules detected, and 100% reproducibility after 8 independent runs was achieved for 10^2^−10^3^ RNA molecules detected.

Therefore, results were in the range observed for previously described RT-LAMP methods detecting all four serotypes in a single reaction [8, 33, 37] with sensitivities between 10 and 100 RNA molecules detected, and RT-LAMP assays distinguishing the serotypes in individual reactions [30, 38]. For the latter assays the analytical sensitivities determined were 10 to 100 plaque-forming units (PFU)/mL and 10 RNA molecules detected respectively. Our RT-LAMP assay for DENV1 showed a limit of detection as per probit analysis of 102 PFU/mL with a confidence interval from 20 to 7.8x10^3^ PFU/mL (data non-shown).

The assays developed were serotype-specific, and no cross-detection of other flaviviruses was observed. Surprisingly, 2 viral preparations tested—KDH0010A (DENV3) and VIMFH4 (DENV4)—were also found positive for DENV1. Subsequent analysis by serotype-specific nested PCR [20] confirmed the presence of DENV1 RNA probably due to contamination during RNA extraction or virus culture, and indicating that the DENV RT-LAMP assays had picked up the contamination correctly.

EQA panels have been developed in order to evaluate the performance and reliability of current diagnostic methods in laboratories worldwide, by using different samples (both negative and positive samples, including different concentrations) that provide information about their specificity and sensitivity [58, 59]. The EQA panel used in this study, provided by QCMD, comprises strains for the 4 DENV serotypes, as well as negative samples. The analysis showed that our RT-LAMP assays passed all the samples included in the 2015 DENV EQA panel, consisting of 8 core and 2 educational samples.

For evaluation with clinical material, RNA was extracted from whole blood samples collected in Tanzania, confirmed as DENV2 positive by qRT-PCR. A bead-based extraction protocol was improved and, in addition, instead of using 50 μL whole blood and eluting in 200 μL RNA, the extraction commenced from 100 μL whole blood and RNA was eluted into 100 μL. Due to this improved extraction protocol, time to positivity reduced from 81–89 min to 55–77 min.

In some cases, there were disparate results between RT-LAMP and qRT-PCR. Sample 1232, negative by RT-LAMP, had a C_T_ value of 28.78, and samples 1241 and 1473, with C_T_ values of 24.27 and 29.13, showed current mean T_T_ values of 70 and 73.9 min, respectively. These differences in results observed may not be related to the sensitivity levels of the individual assay and we suggest that the performance of isothermal amplification reactions could be compromised when not using fresh samples, as previously described [53].

All 11 serum samples collected by Institut Pasteur in Dakar (2014), tested positive by qRT-PCR and the DENV1 and DENV2 RT-LAMP assays. While 3 of the samples could not be characterised with the qRT-PCR protocol, they were successfully amplified by the DENV1 RT-LAMP, providing evidence that determination of serotype is possible when handling samples that have not been serotyped yet.

Based on the results obtained for the fever study in Tanzania, our DENV2 RT-LAMP scored a sensitivity of 95.8% (CI: 0.79–1.00) and specificity of 100% (CI: 0.63–1.00) in reference to the qRT-PCR used by the Swiss Tropical and Public Health Institute, indicating that all detected as positive by the LAMP assay were truly positive and no false positives were detected.

We used predried tubes of RNAstable for shipment of DENV4 and DENV3 RNA extracts from Institut Pasteur du Cambodge. The efficiency of this type of shipment at ambient temperature was disappointing. Surprisingly DENV3 sample RNA extracts suffered most from this type of shipment and this could not be improved in altogether three shipment trials. The results for DENV4 samples indicate specific detection which does not quite match the qRT-PCR sensitivity. DENV3 samples were detectable but sensitivity could not be assessed.

The determination of clinical sensitivity, specificity, PPV and NPV allows interpretation of diagnostic results for clinical decisions [60, 61]. The scores obtained for specificity, sensitivity, PPV and NPV were in the range observed for previously published assays [8, 30, 33, 36–38].

The scores obtained for PPV and NPV estimate the probability that the disease is present or absent depending of the result is positive or negative. Since the samples were collected in a fever study, the results obtained with the RT-LAMP (PPV = 100% and NPV = 85.7%) highlight a good performance of the method in determining true positive cases while excluding negative cases. PPV and NPV are very dependent of the number of positive and negative samples used, providing valuable information during naturally occurring infections in prospective trials. The values obtained in our study may not reflect this as only thirty samples were analysed and a larger number of both positive and negative samples would be needed to refine these results.

To conclude, we have shown a novel approach to designing LAMP primers that makes use of fast growing sequence databases. During the study time the number of complete DENV genome entries grew by 932 genomes deposited. To be able to cover all of the diversity documented, our approach devised 4 complicated mixes of oligonucleotides for the detection of the individual DENV1-4 serotypes. The DENV1 and DENV2 assays were validated with viral RNA extracted clinical samples and showed very good performance parameters. Finally the combination of PCA analysis and molecular detection assays design should also be considered for other molecular assay formats since the available sequence dataset of several pathogens has increased beyond what can be handled by traditional design based on simple alignments.

## Supporting information

S1 File Developed protocol for each DENV RT-LAMP assay. (DOCX)

S2 File STARD 2015 checklist. (DOCX)

S1 Fig RNA standard curve developed to quantify DENV samples by absolute one-step qRT-PCR. (TIF)

S2 Fig PCA and phylogenetic clustering of 1,145 DENV1 genomes. Twenty-one subgroups were necessary to describe all clusters found (variation explained by first, second and third principal component, 47.7%, 11.3% and 9.1% respectively).
(TIF)

S3 Fig PCA and phylogenetic clustering of 477 DENV2 genomes. Twenty subgroups were necessary to describe all clusters found (variation explained by first, second and third principal component, 55.4%, 8.8% and 5.4% respectively).
(TIF)

S4 Fig PCA and phylogenetic clustering of 376 DENV3 genomes. Fifteen subgroups were necessary to describe all clusters found (variation explained by first, second and third principal component, 51.5%, 14.5% and 6.7% respectively).
(TIF)

S1 Table Detailed primers to detect DENV1 by RT-LAMP. (CSV)

S2 Table Detailed primers to detect DENV2 by RT-LAMP. (CSV)

S3 Table Detailed primers to detect DENV3 by RT-LAMP. (CSV)

S4 Table Detailed primers to detect DENV4 by RT-LAMP. (CSV)
