# Supplementary material for: Development and validation of four one-step real-time RT-LAMP assays for specific detection of each dengue virus serotype
Source: PLoS Negl Trop Dis. 2018 May 29;12(5):e0006381. doi: 10.1371/journal.pntd.0006381 (PMC5973574; doi:10.1371/journal.pntd.0006381)
Supplement: S1 File — (DOCX) [file pntd.0006381.s001.docx]

Real time RT-LAMP:

| Temperature (ºC) | Incubation time (min) | | | |
| --- | --- | --- | --- | --- |
|  | DENV1 | DENV2 | DENV3 | DENV4 |
| 64 | 45 | 90 | 75 | 50 |

Prepare the following master mix per LAMP reaction.

|  | Volume (µL) | Final concentrations/amounts |
| --- | --- | --- |
| 10x RM Trehalose | 2.5 | 1x |
| 100 mM MgSO_4_ | 1.5 | 6 mM |
| 25% PEG | 5 | 5% |
| Primer mixes | * | 50 nM F3 and B3  400 nM FIP and BIP  200 nM FLOOP and BLOOP |
| Fluorochrome Dye | 1 |  |
| *Bst*2.0 DNA polymerase (8U/µL) | 1 | 8U |
| Transcriptor Reverse Transcriptase (20U/µL) | 0.5 | 10U |
|  | Up to 24 µL |  |

* Volume dependent of the assay and individual primer concentrations.

**IMPORTANT**: before adding the enzymes (*Bst*2.0 DNA polymerase and Transcriptor RT), heat the master mix at 95 ºC for 5 min, immediately transfer the tube to ice for 5 min (this will melt possible primer multimers, and therefore will increase the specificity of the reaction).

Spin down the tube and add now the enzymes. Split the master mix in the different 0.2 ml tubes or strips.

Add 1 µL nuclease-free water to the negative control sample (NTC) and 1 µL template RNA to the sample tubes.

Note that the volume of template/water added per reaction can also be modified, but considering a final volume of 25 µL
