# Supplementary figures and images for: Development and validation of four one-step real-time RT-LAMP assays for specific detection of each dengue virus serotype
Source: PLoS Negl Trop Dis. 2018 May 29;12(5):e0006381. doi: 10.1371/journal.pntd.0006381 (PMC5973574; doi:10.1371/journal.pntd.0006381)

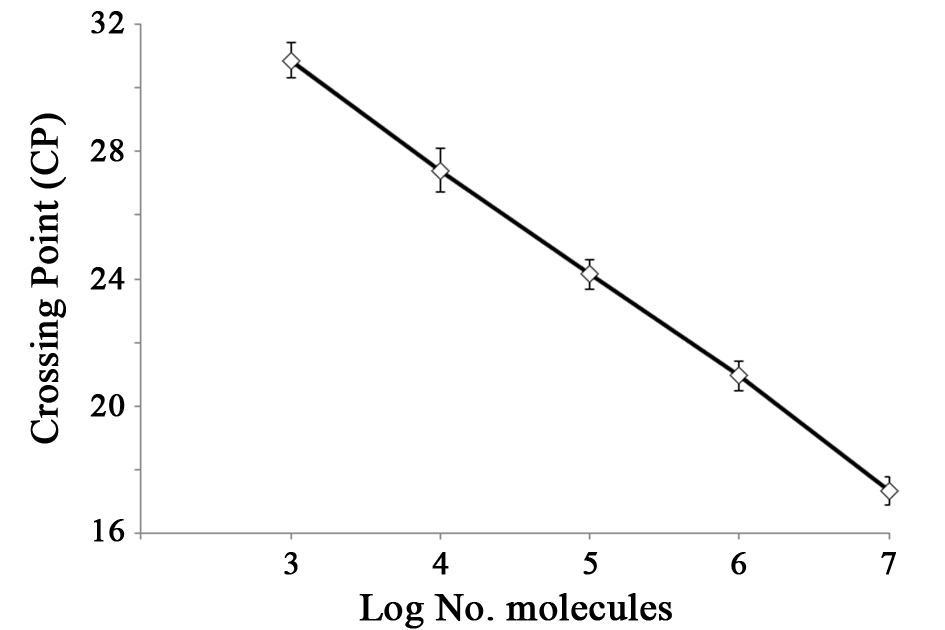

Supplement: S1 Fig — (TIF) [file pntd.0006381.s003.tif]

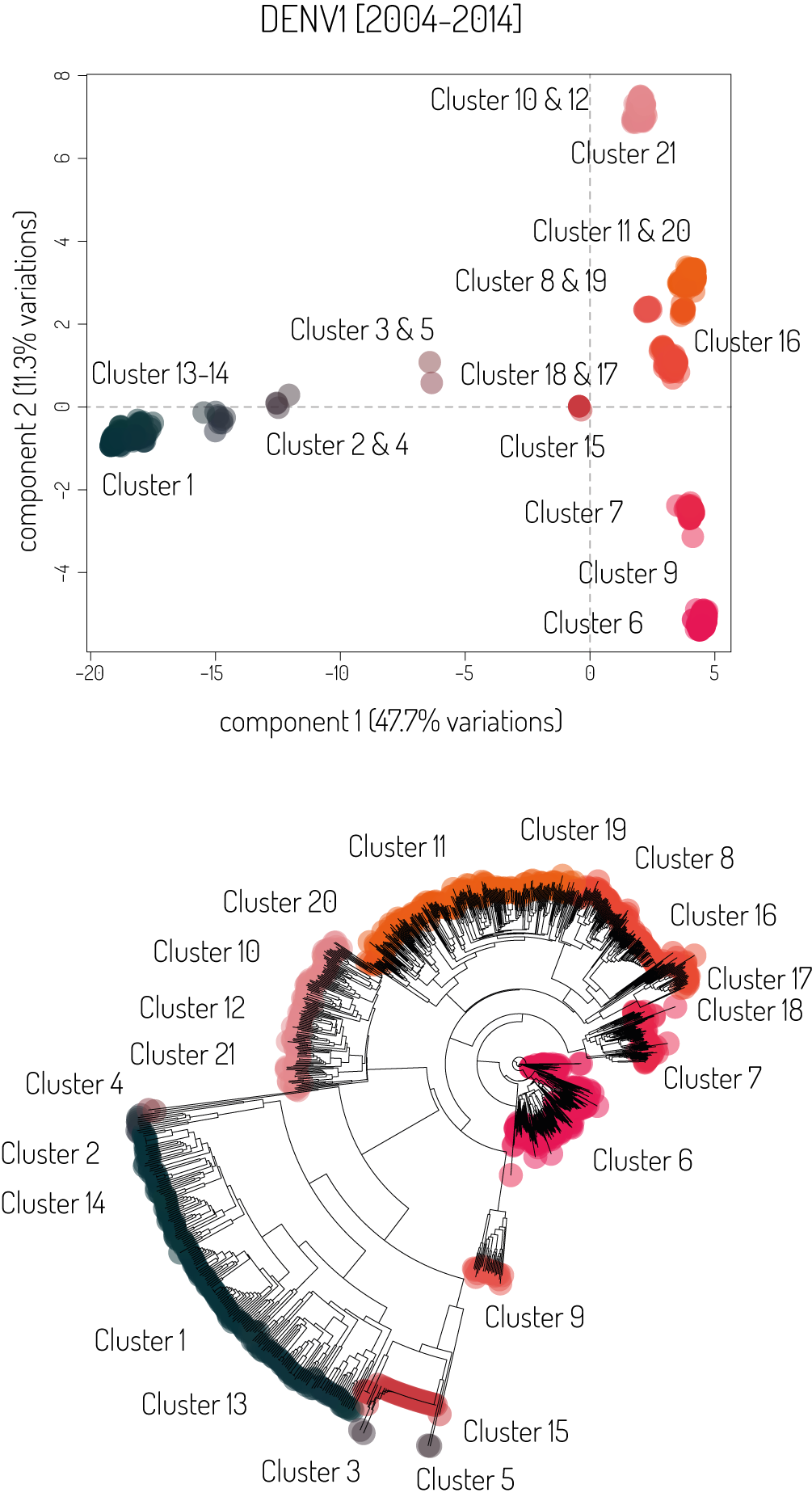

Supplement: S2 Fig — Twenty-one subgroups were necessary to describe all clusters found (variation explained by first, second and third principal component, 47.7%, 11.3% and 9.1% respectively). (TIF) [file pntd.0006381.s004.tif]

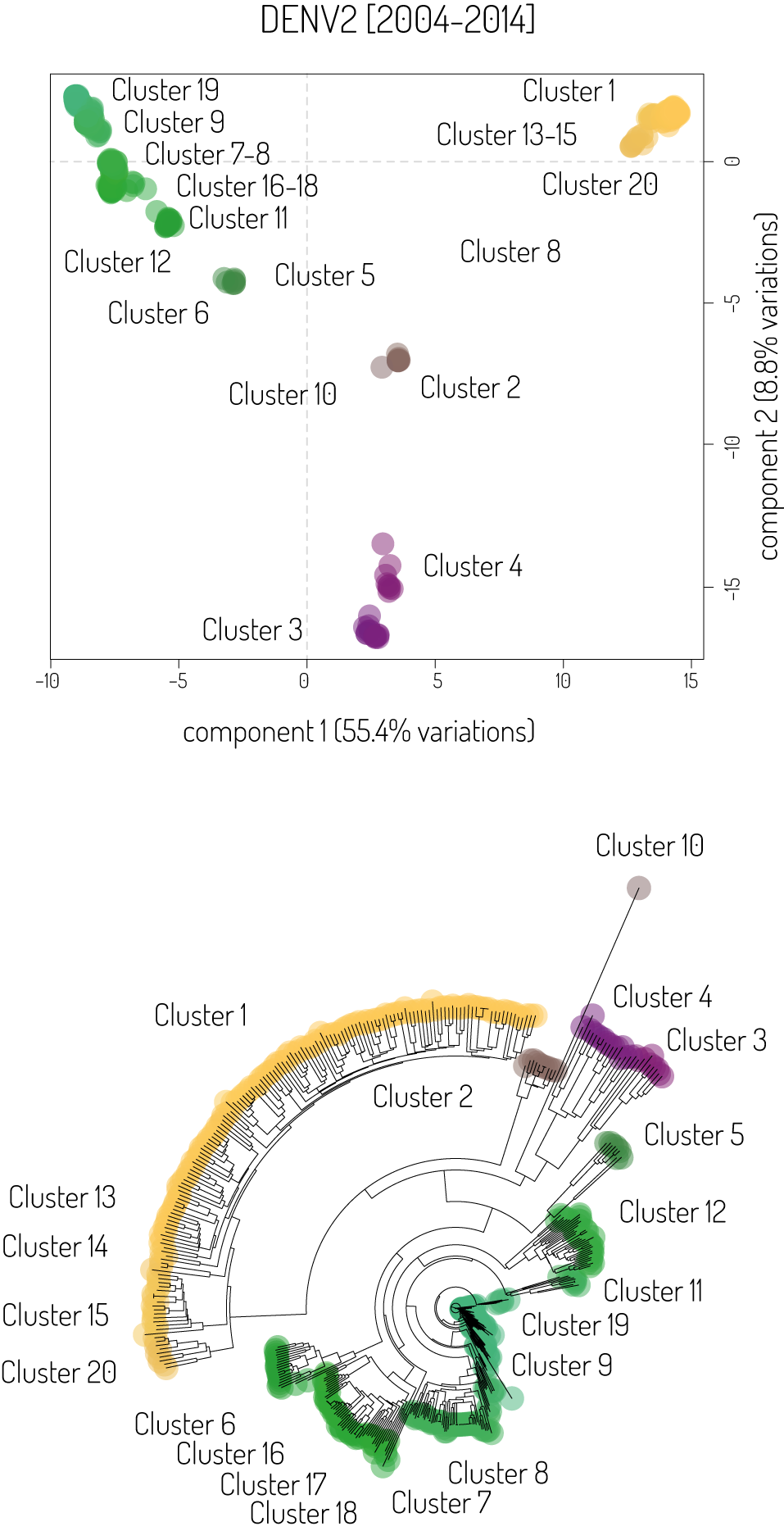

Supplement: S3 Fig — Twenty subgroups were necessary to describe all clusters found (variation explained by first, second and third principal component, 55.4%, 8.8% and 5.4% respectively). (TIF) [file pntd.0006381.s005.tif]

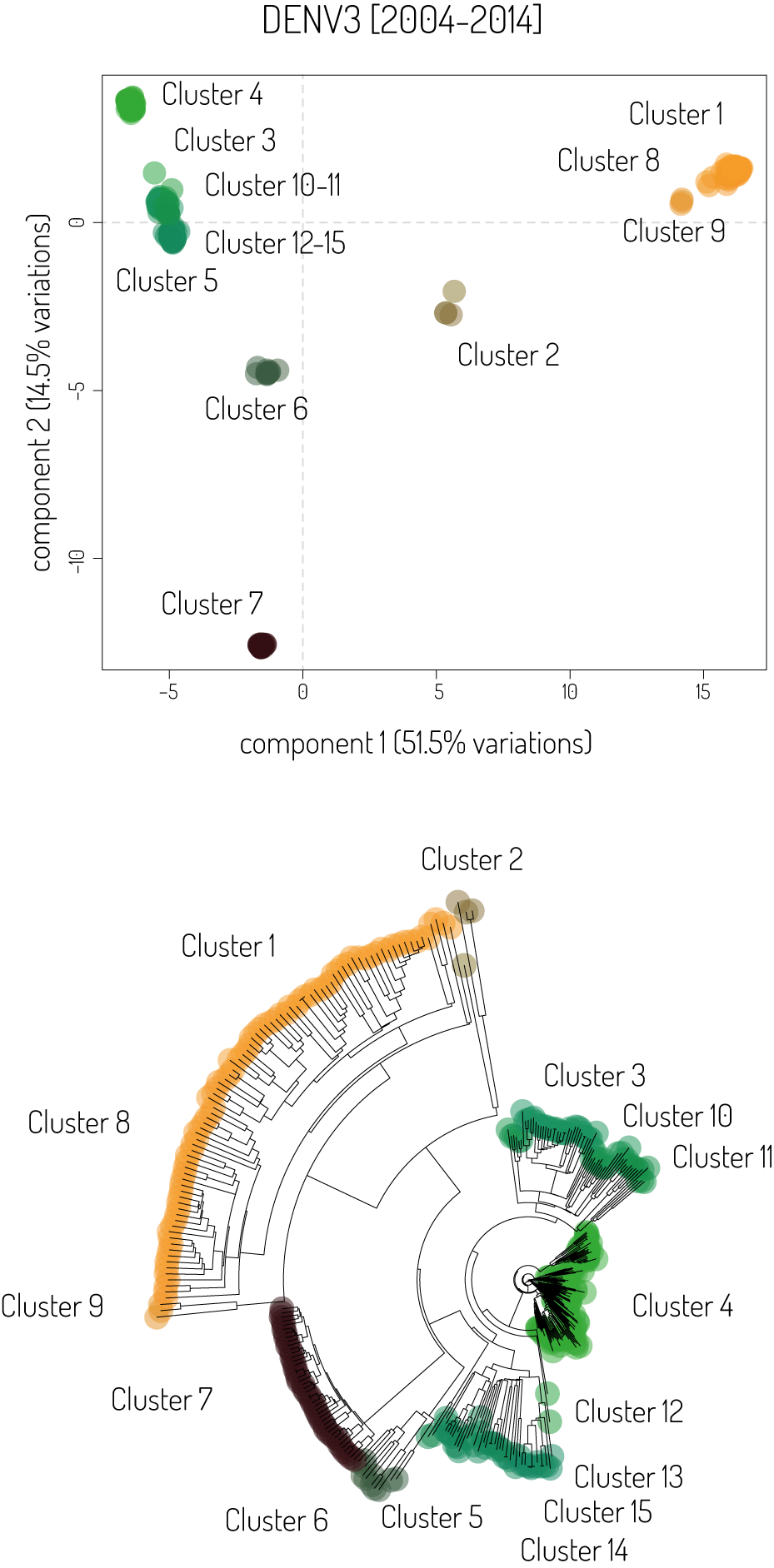

Supplement: S4 Fig — Fifteen subgroups were necessary to describe all clusters found (variation explained by first, second and third principal component, 51.5%, 14.5% and 6.7% respectively). (TIF) [file pntd.0006381.s006.tif]
